# Evolving hard problems: Generating human genetics datasets with a complex etiology

**DOI:** 10.1186/1756-0381-4-21

**Published:** 2011-07-07

**Authors:** Daniel S Himmelstein, Casey S Greene, Jason H Moore

**Affiliations:** 1 Department of Genetics, Dartmouth Medical School, One Medical Center Drive, Lebanon, NH 03756, USA; 2 Lewis-Sigler Institute for Integrative Genomics, Princeton University, Carl Icahn Laboratory, Princeton, NJ 08544, USA

## Abstract

**Background:**

A goal of human genetics is to discover genetic factors that influence individuals' susceptibility to common diseases. Most common diseases are thought to result from the joint failure of two or more interacting components instead of single component failures. This greatly complicates both the task of selecting informative genetic variants and the task of modeling interactions between them. We and others have previously developed algorithms to detect and model the relationships between these genetic factors and disease. Previously these methods have been evaluated with datasets simulated according to pre-defined genetic models.

**Results:**

Here we develop and evaluate a model free evolution strategy to generate datasets which display a complex relationship between individual genotype and disease susceptibility. We show that this model free approach is capable of generating a diverse array of datasets with distinct gene-disease relationships for an arbitrary interaction order and sample size. We specifically generate eight-hundred Pareto fronts; one for each independent run of our algorithm. In each run the predictiveness of single genetic variation and pairs of genetic variants have been minimized, while the predictiveness of third, fourth, or fifth-order combinations is maximized. Two hundred runs of the algorithm are further dedicated to creating datasets with predictive four or five order interactions and minimized lower-level effects.

**Conclusions:**

This method and the resulting datasets will allow the capabilities of novel methods to be tested without pre-specified genetic models. This allows researchers to evaluate which methods will succeed on human genetics problems where the model is not known in advance. We further make freely available to the community the entire Pareto-optimal front of datasets from each run so that novel methods may be rigorously evaluated. These 76,600 datasets are available from http://discovery.dartmouth.edu/model_free_data/.

## Background

Advances in genotyping technologies are changing the way geneticists measure genetic variation. It is now technologically feasible to measure more than one million variations from across the human genome. Here we focus on SNPs, or single nucleotide polymorphisms. A SNP is a single point in a DNA sequence that differs between individuals. A major goal in human genetics is to link the state of these SNPs to disease risk. The standard approach to this problem is to measure the genotypes of people with and without a disease of interest across hundreds of thousands to millions of SNPs. Each of these SNPs is then tested individually for an association with the disease of interest. The goal is to discover SNPs that reliably predict disease susceptibility across many samples [1,2]. This approach has had limited success and the discovery of robust single SNP associations has been difficult to attain [3-5]. Even in the cases where single SNP associations have validated in independent samples, the SNPs often cannot be combined into effective classifiers of disease risk [6]. These studies, by only examining the association of single SNPs, ignore complex interactions that may be critical to understanding disease susceptibility.

The term for complex gene-gene interactions that influence disease susceptibility is epistasis. It is now recognized that studies which ignore epistasis may neglect informative markers [7-10]. Furthermore, epistasis is thought to play a critical role in the understanding of disease because of the complexity present in cellular and biological systems [11] and because it has been well characterized for other complex traits [7,12]. Detecting and characterizing epistasis in all but small datasets is difficult. Examining gene-disease relationships in the context of epistasis requires the consideration of the joint effect of SNPs, and an exhaustive analysis of all possible interactions requires the enumeration of every potential set of SNPs [13]. When datasets contain many SNPs, methods which evaluate each possible combination are not feasible [14].

In human genetics we have, therefore, been confronted by a chicken and egg problem. We believe that it is likely that complex interactions occur, but without methods to detect these interactions in large datasets, we lack the ability to find them. Without found and validated interactions that lead to disease we lack the ability to test new methods on actual genetic datasets. Thus far the problem has been approached with datasets simulated according to hypothetical genetic models as in Velez et al. [15] and Greene et al. [10] among many others. Methods are tested for their ability to find a disease model placed in the datasets. This approach is useful but limited by the diversity and representativeness of the genetic models. The work we present here uses an evolution strategy to generate datasets containing complex genetic interactions that lead to disease without imposing a specific genetic model on the datasets.

### Evolution Strategies

Evolution strategies are algorithms modeled after natural evolution. The combination of several key evolutionary concepts, such as natural selection, population sizes, and mutation rates, produces algorithms capable of finding sought after members of a solution space [16]. Multiple generations allow evolution strategies to direct their results towards an ideal solution by preserving beneficial mutations. One key difference between mutation driven strategies, like the one implemented in our method, and genetic algorithms is the absence of recombination [17]. Recombination, the computational equivalent of genetic crossover, creates new individuals in a population by combining the characteristics of multiple members of the previous generation. Since recombination relies on the exchange of discrete blocks of information, crossover is only appropriate when clear building blocks blueexist [18]. Here it is unclear whether a building block would be a set of individuals or a set of SNPs. Additionally, incorporating building blocks may bias the algorithm to produce a certain subset of gene-disease models unless the building blocks are universally and equally present across the solution space of possible etiologies. Therefore, because it is unclear what the proper building blocks would be, we do not use recombination. Evolutionary algorithms that lack recombination have proven themselves equally as powerful in certain instances and remain able to solve complex problems [19]. For the purpose of our study, we are faced with the challenge of evolving a difficult problem to solve, namely, datasets that have a high-order interaction with no or few lower order effects. Previously, others have used evolutionary algorithms to create problems that are hard for a specific heuristic to solve [20-22]. One novelty of the present study is our use of evolutionary algorithms to find problems without mandating a specific search algorithm or model.

### Multi-objective Optimization and Pareto Optimality

Multiobjective problems maximize or minimize two or more, often competing, characteristics of solutions. Evolutionary algorithms have been used to solve multi-objective optimization problems for more than forty years [23-27]. These strategies are well suited for multi-objective problems because the population can carry out a search with solutions that succeed for different objectives [28,29]. The drawback of this approach is that assigning a single fitness score that encompasses every objective is difficult. Effectively using linear combinations of the objective scores for each objective requires knowledge about the problem and the fitness landscape which is unlikely to be available before a thorough analysis is performed. It is possible, however, to optimize many objectives without *a priori *knowledge by considering non-dominated (i.e. Pareto optimal) solutions as highly fit individuals. A non-dominated solution is one for which there is no solution that is better in all objectives. Here we use an approach focused on Pareto optimal solutions similar to one described by Goldberg [27]. In our approach, we use all Pareto optimal solutions as parents for the next generation, which would be equivalent to using only rank 1 individuals from Goldberg's approach. With this strategy we can explore the Pareto front of solutions which optimize each of our many objectives. We can then provide a number of model free datasets which are optimal with respect to our distinct objectives from a single run of the algorithm.

### Multifactor Dimensionality Reduction (MDR)

**Figure 1:**
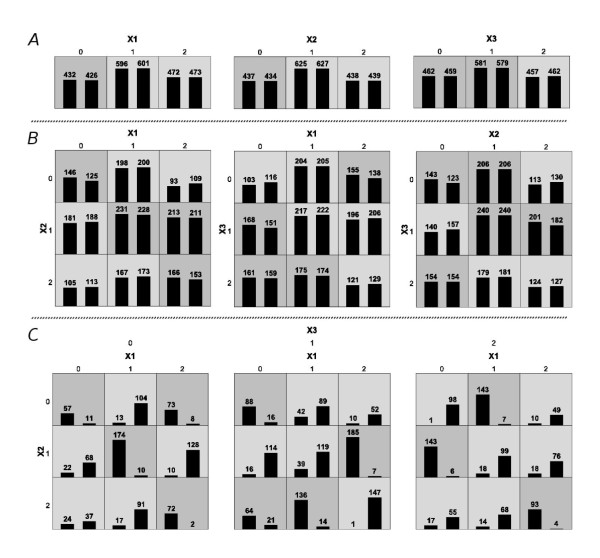
**Display of the MDR Results for a Three-SNP Interaction**. This figure illustrates the solution dataset for a run of our algorithm which attempted to create a three-marker dataset with a high third-order gene-disease association and no lower-level effects. Each square in the plots represents a specific genotypic combination. Within each square the first bar measures the number of cases and the second bar measures the number of controls. The darker squares represent a genotypic combination that was considered high-risk due to the greater number of cases than controls contained within. The top panel, labeled A, shows the relation between each single marker and case-control status. The ability of our algorithm to minimize first-order associations is visible by the relatively equal height of the bars within each square. Of the three one-way associations, X1 versus case-control status scored the highest with an accuracy of 0.502. The middle panel, labeled B, shows the relation between all three two-locus combinations and disease. Again our algorithm succeeded in preventing any major ability to classify disease status based on a specific genotypic combination. The highest two-way effect was between X1, X2 and disease with an accuracy of 0.513. The bottom panel, labeled C, shows the subjects fully decomposed into all genotypic combinations illustrating the third-order effect. Under this level of analysis, each genotypic combination expresses great ability to differentiate between cases an controls. As desired, the accuracy was high at 0.804.

Multifactor Dimensionality Reduction (MDR) is a widely used and a powerful model free method to detect and model gene-gene interactions associated with disease [30,31]. The MDR algorithm and application is highly customizable. This description will focus on our specific implementation of the software and therefore assumes data with an equal number of cases and controls. When evaluating a marker or combination of markers for association to disease status, MDR divides the data into every possible genotypic combination. MDR then classifies each genotypic possibility as high-risk or low-risk depending on whether a given combination of genotypes contains more cases or controls. By dividing the number of subjects that the classification succeeded for by the total number of subjects, MDR produces a measure of association called an accuracy. Accuracies range from 0.5 for instances in which all genotypic combinations contain an equal number of cases and controls to 1 for instances in which any genotypic combination contains either only cases or only controls. Figure 1 provides a visual representation of the MDR algorithm illustrating how the data can be divided by genotype to show differential expression of disease if existent. MDR is well-suited for our application because the accuracies it produces are not biased towards certain gene-disease models. This property arises since the algorithm does not favor accurately predicting cases over controls. Additionally, every subject classified correctly, regardless of its genotypic combination, contributes equally to the resulting accuracy. By combining MDR's agnostic method of computing accuracies with an evolution strategy, we simulate gene-disease models that express the breadth of potential models due to the random nature of our mutation. In essence models are randomly selected and survive based on the sole criteria that they succeed at reaching the desired MDR accuracies. In this way we can generate datasets in a model free manner indicating not that the resulting datasets lack a specific pattern of gene-disease relationship but instead that during the generation process our method is free from mandating specific model choices or restricting our solution space to a subset of models possible under the desired combination of MDR accuracies.

## Results and Discussion

Our parameter sweep of mutation rates showed that, for this problem, a mutation rate of 0.004 led to the greatest success for datasets of 500 people and 0.002 led to the greatest success for datasets of 1000 subjects. Large scale parameter sweeping with the sample sizes that we wished to generate was computationally infeasible, but because the optimal mutation rate was related to the sample size we estimated that for the situation where we wish to evolve datasets containing 3000 subjects with a complex gene-disease relationship, a mutation rate of 0.001 would work, although because of the indirectness required to arrive at this estimate it is not necessarily optimal.

**Figure 2:**
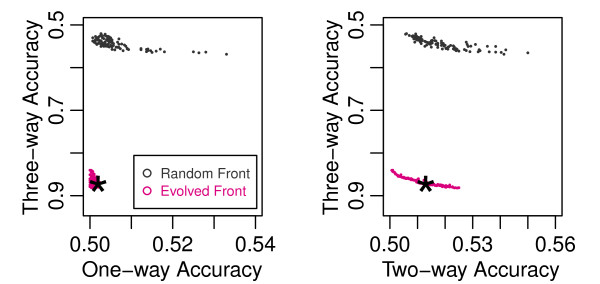
**Final Pareto Fronts of the Random Search and Evolution Strategy**. This figure shows two dimensional projections of the three dimensional Pareto front obtained at the end of one run of the evolution strategy and the Pareto front obtained by randomly generating two million datasets. The three-way accuracy, which is maximized, is plotted against the one and two-way accuracies, which are minimized. The star shows the single dataset from the front chosen as the result of the run, which is used to compare across all runs in Table 1.

We compared the results from our evolution strategy to a random search. The results are presented in Figure 2 and Table 1. Figure 2 shows the Pareto front generated during a single evolved run and the Pareto front generated by a random search over the same number of datasets. It is clear that solutions in the Pareto front from the evolved run are much better than the randomly generated datasets. As Table 1 and Figure 2 show, the evolution strategy consistently outperforms the random search. Furthermore, as Table 1 shows, we were able to consistently generate datasets with a complex gene-disease relationship that lack low-order predictors. In each case the differences between the Pareto front from two million random datasets and that obtained at the end of our evolution strategy was highly significantly different (*p *< 0.001) indicating that these differences are not due to chance. By generating our datasets through an evolution strategy not wedded to any specific model, we achieved datasets that represent the multiplicity of models possible under the desired attribute construction of high-order interaction without low-order interaction.

**Table 1:** A Summary of the Accuracies Obtained for the Evolution Strategies and Random Search

	Parameters				Results		
	
**Gen**.	**Pop**.	HW	One-way(sd)	Two-way(sd)	Three-way(sd)	Four-way(sd)	Five-way(sd)
1	2000000	No	↓0.506 (0.006)	↓0.518 (0.009)	↑0.543 (0.012)		
2000	1000	No	↓0.502 (0.001)	↓0.511 (0.007)	↑0.886 (0.023)		
2000	1000	Yes	↓0.504 (0.002)	↓0.509 (0.003)	↑0.680 (0.024)		

1	2000000	No	↓0.507 (0.004)	↓0.519 (0.006)	-	↑0.571 (0.011)	
2000	1000	No	↓0.502 (0.001)	↓0.510 (0.003)	-	↑0.897 (0.018)	
2000	1000	Yes	↓0.507 (0.003)	↓0.513 (0.003)	-	↑0.673 (0.009)	
1	2000000	No	↓0.507 (0.004)	↓0.519 (0.005)	↓0.536 (0.008)	↑0.567 (0.010)	
2000	1000	No	↓0.501 (0.000)	↓0.504 (0.001)	↓0.518 (0.003)	↑0.750 (0.015)	

1	2000000	No	↓0.507 (0.004)	↓0.520 (0.005)	-	-	↑0.612 (0.011)
2000	1000	No	↓0.502 (0.001)	↓0.510 (0.002)	-	-	↑0.895 (0.009)
2000	1000	Yes	↓0.511 (0.003)	↓0.518 (0.003)	-	-	↑0.693 (0.008)
1	2000000	No	↓0.508 (0.003)	↓0.520 (0.004)	↓0.536 (0.006)	↓0.561 (0.007)	↑0.607 (0.010)
2000	1000	No	↓0.503 (0.001)	↓0.508 (0.001)	↓0.518 (0.002)	↓0.543 (0.004)	↑0.690 (0.008)

HW indicates whether or not the datasets were selected based on their conformance to Hardy-Weinberg equilibrium. The two horizontal dividers separate three, four, and five locus datasets. Completely blank cells of the table signify a non-existent measurement. Cells with a dash represent a value that exists but was not measured or selected for in the evolutionary computation. The results panel shows the mean and (standard deviation) of the accuracies across the one-hundred best per run datasets. The arrows appearing before each accuracy indicate whether that level of effect was minimized or maximized during the evolutionary computation. Gray rows represent the results from the random searches.

### Dataset Availability

All Pareto optimal datasets generated during these experiments are available from the website http://discovery.dartmouth.edu/model_free_data/. We provide a number of means of obtaining datasets. First, we provide archive files of "best of runs" obtained with and without Hardy-Weinberg equilibrium constraints. These representative datasets are obtained by choosing the dataset with the smallest euclidean distance between its own values and the optimum values obtained by all datasets on the Pareto front as described in Methods. In addition to these representative datasets, for each run we provide all datasets that, at the end of the run, make up the complete Pareto front. To assist with the use of these datasets we further provide an information file for each run containing the characteristics of every dataset in the Pareto-optimal front. From these files it is possible for investigators to develop suites of datasets that display certain characteristics (e.g. one and two way accuracies less than 52% and four-way accuracies of approximately 70%). Using datasets generated from our provided results, investigators can test novel methods across data exhibiting gene-disease relationships unconstrained by specific genetic models.

Additionally by generating two types of four locus datasets - datasets with minimized three-way accuracies and datasets with unconstrained three-way accuracies - we give researchers the flexibility to pick datasets applicable to their specific study. The four and five locus datasets that do not minimize all lower-level effects may be helpful for researchers who wish to test their algorithm on datasets where the etiology contains lower-order marginal effects as part of four or five locus models. The datasets which minimize all lower-level effects will be helpful for evaluating how a search algorithm performs on the most difficult etiologies where meaningful gene-disease relationships do not appear until models embracing the exact number of SNPs involved in the interaction are evaluated. The SNPs we provide can be combined with other SNPs unrelated to disease status to represent a three, four, or five-way genetic interaction in a sea of noisy SNPs.

## Conclusions

Researchers have previously used evolutionary computing to generate the models underlying epistatic (i.e. interaction based) gene-disease relationships for both two-marker [32] and higher [33] relationships. Here we simulated epistatic datasets which are not constrained to specific predetermined models but instead achieve a diversity of gene-disease relationships created by the randomness of mutation and enhanced through the model-agnostic selective power of evolution. Using this strategy we created 76,600 datasets with complex gene-disease relationships, which we made publicly available. These datasets provide test beds for novel genetic analysis methods. By providing human genetics datasets with complex interactions that do not assume a model we hope to bypass the chicken and egg problem that has previously confronted the field. Methods tested on datasets generated in this manner may better generalize to true genetic association datasets.

Future work should focus on evaluating potential building blocks, so that crossover can improve the efficiency of the search. Potential building blocks include subsets of the individuals in each dataset, subsets of the SNPs in each dataset, or subsets of both individuals and SNPs in each dataset. It is not intuitive which approach is most useful, so these options should be fully explored. It may be that customized crossover operators are required to obtain useful genetic mixing for this problem. One worry with incorporating building blocks is that within all possible gene-disease models with similar scoring attributes specific models may be favored by the method for selecting building blocks. To evaluate whether a certain building block would undermine the multiplicity of models generated under the approach of this study, one could evaluate the prevalence of the proposed building block within the final datasets we've generated. If the building block is not universally and equally present within each dataset, then a recombinational approach would exclude a portion of the solution space and is not advisable.

Future work should also focus on making these datasets and others generated in this manner widely available. By dividing the resulting datasets into standardized testing and training datasets, it would be feasible to compare algorithms in a straightforward and objective manner. This comparison of algorithms would provide a great deal of information about these methods to human geneticists attempting to understand the basis of common human disease. By providing open and publicly available datasets which do not assume a model but which contain a complex relationship between individual and disease, we hope to improve our understanding of commonly used methods in human genetics. We also hope to provide a framework for objectively testing future methods. The ability to effectively benchmark methods across a comprehensive evaluation suite of datasets provides an important testing ground to aid the search for uncovering the underlying basis of common human diseases.

## Methods

### A Model Free Dataset Generation Method

In terms of evolution, a dataset from a set of datasets in our study is analogous to an individual in a population. All datasets adhere to a basic structure. Each row represents a subject in a balanced genotypic case-control study. The last column represents whether a subject is a case or control coded respectively as 1 or 0. The preceding columns represent the subjects' genotypes at the associated bi-allelic SNPs which exist in three states coded here with 0, 1 (heterozygous case), or 2. The format of our datasets allows us to compute for each dataset certain attributes which form the selection criteria for our evolutionary strategy. The association of each SNP or combination of SNPs to disease status is measured by computing an MDR accuracy. For each order of marker-disease relationship which we are attempting to minimize, the largest MDR accuracy for that order forms an attribute. Every dataset therefore has an attribute which equals the maximum MDR score for all single SNP to disease associations. Another attribute measures the maximum MDR score for all two-SNP to disease associations in a given dataset. The highest order SNP to disease attribute is the MDR score of the association between all SNPs and disease.

To begin the evolutionary algorithm, we filled our first population with 1000 initialized datasets. Datasets were initialized by equally assigning subjects case or control status. We then randomly assigned genotype values assuming a minor and major allele frequency of 0.5. After each stage of our algorithm with a full population of 1000, we selected the datasets to survive. To accomplish this stage, we selected the Pareto optimal datasets based on their attributes. For example, when aiming to create a dataset with a high third-order interaction and no lower-level effects, we took datasets that were non-dominated in one of more of the following regards: smallest first-order attribute, smallest second-order attribute, and largest third-order attribute. The set of surviving datasets advanced to the next generation and also acted as the parents for new datasets. Each parent was replicated with mutation until the population size of 1000 was full. We performed the replication by randomly mutating the genotype values for each SNP according to our mutation rate of 1 in 1000. We continued this process for 2000 generations resulting in a final set of Pareto optimal datasets. Using the method described above, we ran the evolutionary strategy 100 times selecting for a high third-order effect and no lower-level effects, 100 times selecting for a high fourth-order effect and no one or two-way effects, and 100 times selecting for a high fifth-order effect and no one or two-way effects.

In addition to the datasets just described, we created four and five locus datasets with no lower-level effects. For example on a five locus dataset, one through four-way effects were minimized and five-way effects were maximized. The number of objectives to be optimized during the evolutionary computation increased from three to four or five. Because this adds one or two more dimensions to the Pareto front, it dramatically increased the size of the Pareto front. To ensure that each parent had an opportunity to generate a reasonable number of off spring, we limited the number of parents taken to the next generation to 100 when we were creating the four and five locus datasets with no lower-level effects. When there were more than 100 individuals on the front, we chose the individuals in the "elbow" of the Pareto front (i.e. non-extreme individuals). This tie-breaker kept individuals which were good in regards to more than one dimension at the cost of those which excelled in a single dimension. We chose the Pareto cap of 100 because as a factor of our population size it ensured every dataset an equal number of o spring. In addition, the runs of our algorithms optimizing three attributes had succeeded while evolving under Pareto fronts numbering around 100.

For SNPs not under selective pressure in humans, these states exhibit what is called Hardy-Weinberg equilibrium (HWE). Hartl and Clark provide an excellent overview of the Hardy Weinberg principle [34]. Because most SNPs are not under selection, deviations from HWE have historically been used as a marker of genotyping error [35]. The implicit assumption is that a SNP which is not in HWE is more likely to be a genotyping error than a SNP under selection. Examinations of early genetic association studies suggested that these concerns may be well founded [36]. As genotyping methods improve and genotyping error is reduced, it becomes more likely that these SNPs are under selective pressure and less likely that deviations from HWE are due to genotyping error, and thus it becomes less likely that geneticists will filter SNPs which deviate from HWE. Indeed new methods have been developed which use the principles of Hardy-Weinberg equilibrium to detect an association between a genotype and disease [37]. Because the field is currently in transition we provide two sets of datasets, one set where we optimize for non-significant HWE genotype frequencies and one where we do not. In both cases we have initialized the frequencies of the genotype states as under HWE but selection can alter these frequencies.

By adding the attribute of HWE equilibrium as a Pareto criterion we can generate datasets containing SNPs that would not be filtered by currently used quality control measures. In this way we develop datasets where there is a complex relationship between genotype and disease. With a wide array of datasets we can then test the ability of novel methods to detect and characterize complex epistatic relationships without making assumptions about the underlying genetic model. Because the result of each run is a set of Pareto optimal solutions, users can pick solutions with a wide array of difficulties to use while evaluating novel methods. For the set of results where we attempted to preserve Hardy-Weinberg equilibrium we actually minimize disequilibrium. Specifically we minimize the chi-square statistic which measures deviation from HWE. Selecting for the additional objective of adherence to HWE requires limiting the Pareto front to the 100 most dominating non-dominated datasets as we did with the four locus datasets with no one through three-way effects.

At the conclusion of each run we have a front of Pareto optimal datasets. Because comparing entire Pareto fronts is difficult we wish to provide, in addition to the front, a single member of the Pareto-optimal group which can represent the run. We have done this by picking the individual dataset with the smallest euclidean distance from the best values obtained for each measure.

### Experimental Design and Analysis

**Figure 3:**
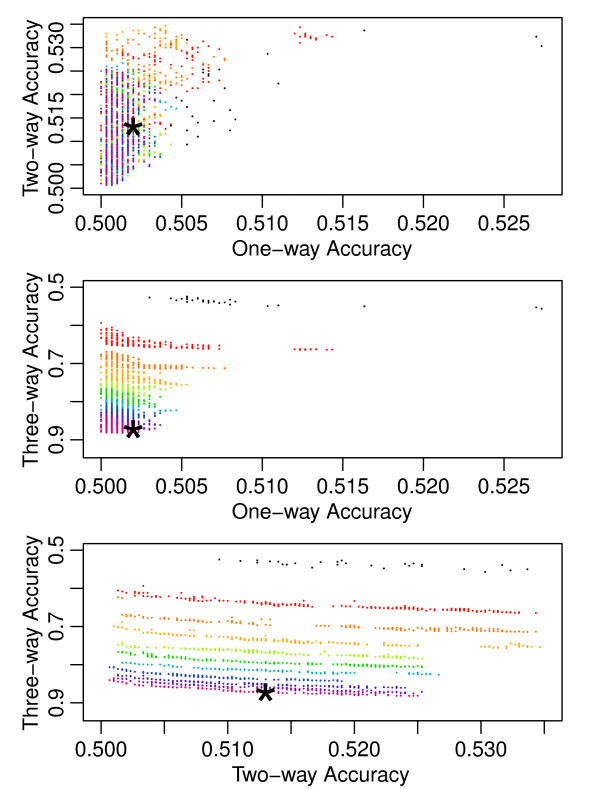
**Progress of the Pareto Fronts over Thousands of Generations**. This figure maps the progress of one run of the three-way algorithm across the 2000 generations of the evolution strategy. Instead of a single three-dimensional graph, we decomposed the illustration into three pairwise plots in which each solution dataset drawn appears once on each plot. Each dot represents a dataset from a Pareto front and shows how that dataset scored on the x and y-axis attributes. The axis are drawn so points closer to the bottom-left corners of the plots represent more optimized solutions. The black dots represent the non-dominated solutions from the original random initialization of 1000 datasets. The Pareto fronts from every subsequent two-hundredth generation are drawn and assigned a color based on their generation. The chronological generation progression follows the colors of a rainbow and can be most easily discerned from the bottommost plot. The star indicates the dataset that was chosen from the final Pareto front to represent the run. These datasets are taken from each run, according to the euclidean distance strategy discussed in the Model Free Dataset Generation Method section, and used to calculate the summary statistics in Table 1. This figure provides insight into the difficulty of the problem. Minimizing the one and two-way accuracies occurs relatively quickly (within the first few hundred generations). Maximizing the higher order accuracies continues throughout the entire run with progress continuing into the two-thousandth generation.

To implement our evolution strategy, we first determined a useful mutation rate. Because our method for generating datasets was driven by mutation, we needed to employ an effective mutation rate to evolve solutions with our sought-after attributes. Additionally, by finding an optimal mutation rate, we could reduce the computation time (number of generations) needed to arrive at a given quality of solution or alternatively increase the quality of solution for a given time of computation. We examined algorithm effectiveness across the mutation rate landscape by evaluating rates of 0.05, 0.04, 0.03, 0.02, 0.01, 0.008, 0.006, 0.004 and 0.002 for sample sizes of 500 and 1000 using a fixed number of generations (750) and a fixed population size (1000). We then used the four-way testing accuracy to evaluate how far the evolution had progressed. We used these results to pick an appropriate mutation rate for the number of generations and population size. We terminated our evolutionary strategy at 2000 generations due to computational time constraints and the diminishing marginal returns from each additional generation which can be seen in Figure 3. The population size of 1000 provided sufficient sampling of the Pareto front while remaining computationally feasible.

To determine whether our Pareto evolution strategy outperformed a random search, we generated two million random datasets and compared the resulting Pareto front to the Pareto fronts generated at the end of evolution in our system. We tested the significance of the differences observed for the accuracies from the fronts from evolved runs and those from the randomly generated runs. We statistically tested these differences with Hotelling's T-test and considered the differences significant when the p-value was less than or equal to 0.05. We chose the standard 0.05 p-value cutoff because a one in twenty chance of mistakenly finding the results significant seemed acceptable. Additionally however, the p-value need not be solely understood in the context of a significance cutoff. For example, if the resulting p-values are extremely small, we can conclude significance at cutoffs more stringent than 0.05. Therefore, if the p-values from our Hotelling's T-test are several orders of magnitudes smaller than 0.05, our choice of 0.05 compared to other plausible significance cutoffs is irrelevant.

Our final task was to generate sets of Pareto optimal datasets each exhibiting three-way interactions, four-way interactions and five-way interactions. In each case we maximized three, four, and five-way MDR accuracies respectively while minimizing one and two way accuracies. We generated two additional sets of Pareto optimal datasets that contained four and five-way interactions and minimized lower-level effects. We further generated datasets both under pressure to maintain Hardy-Weinberg equilibrium and irrespective of HWE. For each parameter setting (three, four, and five-way interactions with and without HWE and four and five-way interactions with no lower-level effects) we generated 100 sets of datasets for a total of 800 sets of Pareto optimal datasets. In total we have generated more than 70,000 datasets with a complex gene-disease relationship and made these datasets available to researchers as described in the Dataset Availability section.

Our decision to produce three, four, or five-way interacting SNP datasets as opposed to datasets with two SNPs or more than five SNPs was guided by several factors. While datasets with a two-way interaction and no main effects are useful, several simulations with this attribute construction already exist [15,32]. Our study is tailored towards researchers who desire a currently sparse higher-order class of simulated datasets that are more difficult to solve than existing data simulations. Additionally, with bi-allelic SNPs only nine genotypic combinations exist for any two SNPs. Given this lower complexity of two-SNP interactions [38], the evolutionary strategy and Pareto optimization underlying our simulation presents fewer advantages over other methods of simulation. With fewer possible genotypic combinations, simulating across the breadth of possible etiologies is easier. The computational intensity of evolution strategies for data simulation, which we justify through the difficulty of our objectives, may not be necessary. Conversely, datasets that contain too complex of an etiology lack relevance if their order is so high and structure so complex that search algorithms will be unable to solve them in the near future. Additionally, our tuned algorithm of Pareto optimization operating under specific parameters will struggle with higher-order datasets. Absent an increase in population size within each generation of our evolution strategy, the number of non-dominated solutions may become unwieldy and cripple the algorithm effectiveness as we attempt to optimize increasing attribute numbers. Computational time and resources also provide limits on the order of the datasets we could generate. As SNP number increases, the comparisons computed for the MDR portion of our algorithm grows rapidly as do memory and disk storage space to a lesser extent.

## Competing interests

The authors declare that they have no competing interests.

## Authors' contributions

DSH, CSG, and JHM conceived the study, discussed the approaches, and wrote the manuscript. DSH implemented the method. DSH and CSG performed the experiments. All authors read and approved the final manuscript.
