# Erratum to: Evolving hard problems: generating human genetics datasets with a complex etiology

**DOI:** 10.1186/s13040-016-0085-5

**Published:** 2016-02-03

**Authors:** Daniel S. Himmelstein, Casey S. Greene, Jason H. Moore

**Affiliations:** Department of Genetics, Dartmouth Medical School, One Medical Center Drive, Lebanon, NH 03756 USA; LewisSigler Institute for Integrative Genomics, Princeton University, Carl Icahn Laboratory, Princeton, NJ 08544 USA

## Erratum to

After publication of this article [1], it has been noticed that Figs. 1 and 3 (Figs. 1 and 2 respectively here) had been incorrectly reverted in the original article [1].

**Fig. 1 (Fig. 1 in original article [1]):**
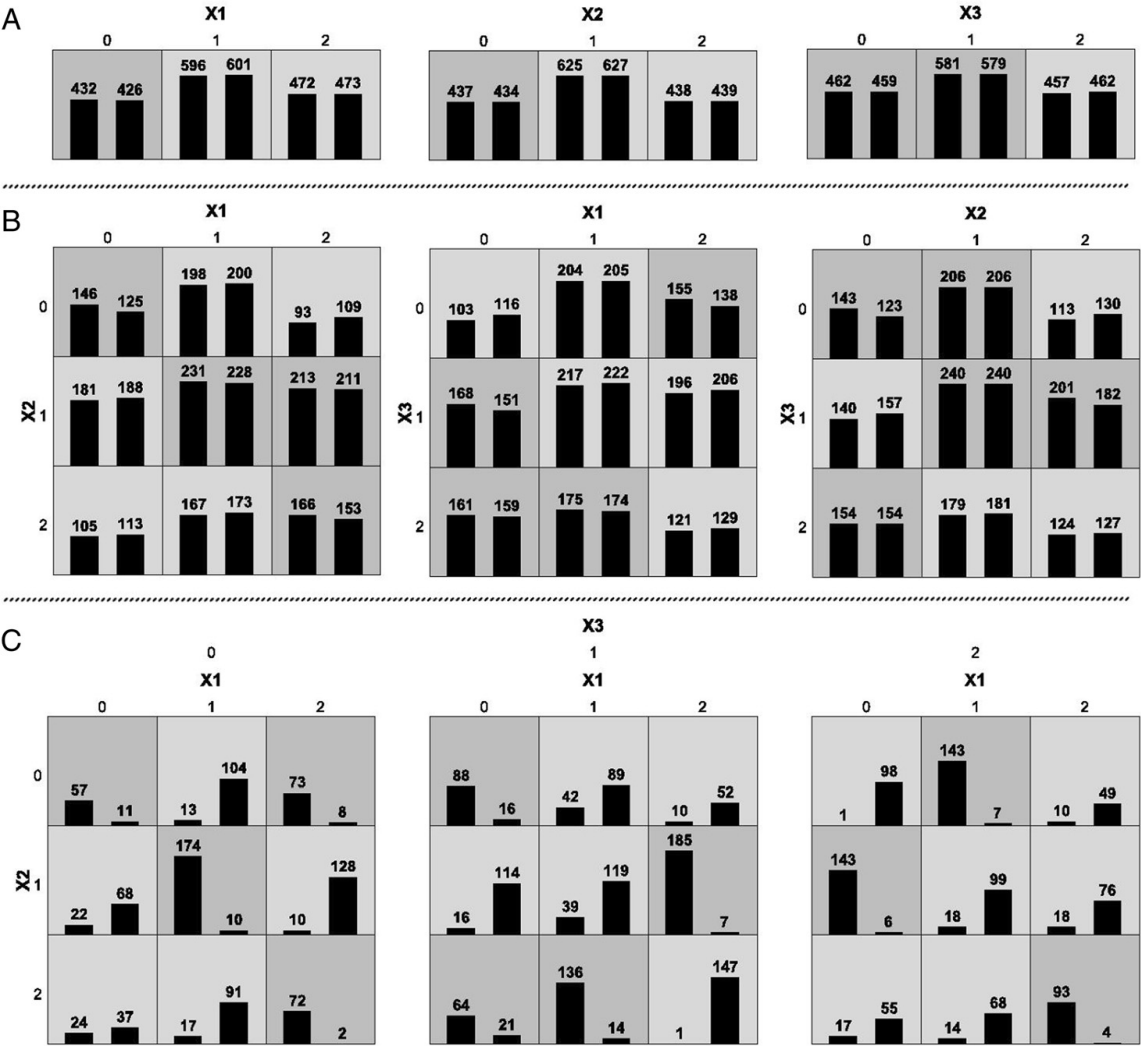
Display of the MDR Results for a Three-SNP Interaction. This figure illustrates the solution dataset for a run of our algorithm which attempted to create a three-marker dataset with a high third-order gene-disease association and no lower-level effects. Each square in the plots represents a specific genotypic combination. Within each square the first bar measures the number of cases and the second bar measures the number of controls. The darker squares represent a genotypic combination that was considered high-risk due to the greater number of cases than controls contained within. The top panel, labeled **a**, shows the relation between each single marker and case–control status. The ability of our algorithm to minimize first-order associations is visible by the relatively equal height of the bars within each square. Of the three one-way associations, X1 versus case–control status scored the highest with an accuracy of 0.502. The middle panel, labeled **b**, shows the relation between all three two-locus combinations and disease. Again our algorithm succeeded in preventing any major ability to classify disease status based on a specific genotypic combination. The highest two-way effect was between X1, X2 and disease with an accuracy of 0.513. The bottom panel, labeled **c**, shows the subjects fully decomposed into all genotypic combinations illustrating the third-order effect. Under this level of analysis, each genotypic combination expresses great ability to differentiate between cases an controls. As desired, the accuracy was high at 0.804

**Fig. 2 (Fig. 3 in original article [1]):**
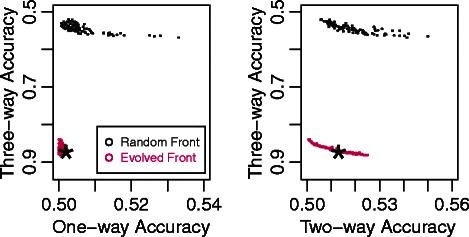
Progress of the Pareto Fronts over Thousands of Generations. This figure maps the progress of one run of the three-way algorithm across the 2000 generations of the evolution strategy. Instead of a single three-dimensional graph, we decomposed the illustration into three pairwise plots in which each solution dataset drawn appears once on each plot. Each dot represents a dataset from a Pareto front and shows how that dataset scored on the x and y-axis attributes. The axis are drawn so points closer to the bottom-left corners of the plots represent more optimized solutions. The black dots represent the non-dominated solutions from the original random initialization of 1000 datasets. The Pareto fronts from every subsequent two-hundredth generation are drawn and assigned a color based on their generation. The chronological generation progression follows the colors of a rainbow and can be most easily discerned from the bottommost plot. The star indicates the dataset that was chosen from the final Pareto front to represent the run. These datasets are taken from each run, according to the euclidean distance strategy discussed in the Model Free Dataset Generation Method section, and used to calculate the summary statistics in Table 1. This figure provides insight into the difficulty of the problem. Minimizing the one and two-way accuracies occurs relatively quickly (within the first few hundred generations). Maximizing the higher order accuracies continues throughout the entire run with progress continuing into the two-thousandth generation

The correct presentation of Figs. 1 and 3 (Figs. 1 and 2 respectively here) are included in this erratum.
